# Real-world treatment patterns and outcomes in metastatic clear cell renal cell carcinoma following immune checkpoint inhibitor progression (2018-2023)

**DOI:** 10.1093/oncolo/oyag326

**Published:** 2026-09-15

**Authors:** Neil J Shah, Rahul Ravilla, Andrew J Osterland, Karen Todoroff, Ching-Yu Wang, Pratik Rane, Hakim Saal, Paul R Conkling, Ding Wang, Robert J Motzer

**Affiliations:** Genitourinary Oncology, Memorial Sloan Kettering Cancer Center, Weill Cornell Medical College, New York, NY 10021, United States; US Oncology Network/New York Oncology Hematology, Albany, NY 12206, United States; Ontada, Boston, MA 02110, United States; Ontada, Boston, MA 02110, United States; Merck Sharp & Dohme LLC, a subsidiary of Merck & Co., Inc., Rahway, NJ 07065, United States; Merck Sharp & Dohme LLC, a subsidiary of Merck & Co., Inc., Rahway, NJ 07065, United States; Formerly of Eisai Inc., Nutley, NJ 07110, United States; Ontada, Boston, MA 02110, United States; Merck Sharp & Dohme LLC, a subsidiary of Merck & Co., Inc., Rahway, NJ 07065, United States; Genitourinary Oncology, Memorial Sloan Kettering Cancer Center, Weill Cornell Medical College, New York, NY 10021, United States

**Keywords:** real-world, immune checkpoint inhibitor, renal cell carcinoma, metastatic, progression post-ICI

## Abstract

**Introduction:**

Immune checkpoint inhibitors (ICI) have become standard of care for renal cell carcinoma (RCC) in the adjuvant, first-line and pre-treated settings. However, treatment patterns and outcomes of patients with RCC that progresses following ICI treatment are not well characterized.

**Methods:**

A retrospective review of electronic health records from a US community oncology network included patients with metastatic clear cell RCC (mccRCC) that progressed after an initial ICI-containing regimen and received subsequent systemic therapy (index) between 01/01/2018 and 01/31/2023. Patient characteristics and treatment patterns were described. Real-world progression-free survival (rwPFS) and overall survival (OS) were analyzed using Kaplan-Meier method by line of therapy (LOT) of the first ICI-containing regimen and index regimen class.

**Results:**

Overall, 299 patients were included (median [IQR] age 68 [60-75] years, 73% male, 85% white, 87% intermediate or poor IMDC risk, median follow-up 11.3 months) with the first ICI-containing regimen initiated in the adjuvant (*n* = 5, 2%), first-line (*n *= 203, 68%) or second-line (*n *= 91, 30%) settings. Vascular endothelial growth factor receptor tyrosine kinase inhibitor (VEGFR-TKI) monotherapy was the most common index (post-IO) regimen class (62% any VEGFR-TKI, 46% cabozantinib). Median (95% CI) rwPFS and OS were 6.0 (5.4, 6.8) and 14.3 (12.1, 16.4) months, respectively. No differences were observed in rwPFS and OS by LOT of the first ICI-containing regimen (*P* = 0.763, *P *= 0.885, respectively) or index regimen class (*P *= 0.721, *P *= 0.747, respectively).

**Conclusion:**

In real-world patients with mccRCC that progressed to the post-ICI setting, VEGFR-TKI monotherapies were the most common post-ICI regimens and differences in clinical outcomes were not observed by LOT of the first ICI-containing regimen or index regimen class. There is high unmet need for novel therapies in the post-ICI setting.

Implications for Practice Although significant progress has been made in the treatment of ccRCC in adjuvant, first and later-line settings, there is an unmet medical need for innovative and novel therapies in the post-ICI space to improve clinical outcomes.

## Introduction

In the last few years, anti-PD-1/PD-L1 immune checkpoint inhibitor (ICI) treatments in combination with cytotoxic T-lymphocyte-associated protein 4 (CTLA-4) checkpoint inhibitor or with vascular endothelial growth factor receptor tyrosine kinase inhibitors (VEGFR-TKIs) have become standard of care for the first-line (1 L) treatment of metastatic clear cell renal cell carcinoma (mccRCC). As compared to the previous standard of care for 1 L (sunitinib), the CheckMate-214 trial demonstrated superior overall survival (OS) of nivolumab+ipilimumab in the IMDC intermediate-poor risk population, whereas the KEYNOTE-426 (pembrolizumab+axitinib), Checkmate-9ER (nivolumab+cabozantinib) and CLEAR (pembrolizumab+lenvatinib) trials demonstrated superior progression-free survival (PFS) and OS of the ICI-based combination regimens.^1–4^ Currently, there are several options for treatment in RCC in 1 L and pretreated setting. However, recommendations for specific regimens depend on various factors, including IMDC risk groups, histology and prior exposure to anti-PD-1/PD-L1 ICI and there is no preferred treatment in the post-ICI setting.^5–10^ In particular, the emergence of different resistance mechanisms to ICI and VEGFR-TKI can limit the durability of treatment benefit underscoring the need to better define optimal treatment sequencing and develop novel therapeutic strategies capable of overcoming resistance.^11^ Other recommended regimens in the post-ICI setting are axitinib, cabozantinib, everolimus+lenvatinib, tivozanib, as well as belzutifan, which is a novel HIF-2a inhibitor.^6^^,^^12–14^ Despite recommendations, most of these regimens were not investigated in phase III clinical trials in the post-ICI setting, with our without prior VEGFR-TKI exposure, whereas belzutifan was the only agent studied in post-ICI and post-VEGFR-TKI setting.^13^

There have been several clinical trials initiated to evaluate additional novel regimens to address the unmet need among patients with mccRCC post-ICI.^15–19^ Although early proof-of-concept studies provided preliminary evidence supporting the use of ICI-TKI combination therapy in the first-line mccRCC setting, negative findings from the CONTACT-03 (atezolizumab+cabozantinib) and TiNivo-2 (tivozanib+nivolumab) studies highlight the lack of clinical benefit of ICI-TKI combination therapy compared to VEGFR-TKI monotherapy in the post-ICI setting, which indicates a need for novel alternatives.^20–24^ Additional studies of existing approved regimens such as everolimus+lenvatinib (LenCabo) and novel combinations such as belzutifan+lenvatinib (LITESPARK-011), belzutifan+cabozantinib (LITESPARK-003) and casdatifan+cabozantinib (PEAK-1) are recently published or ongoing.^25–28^

Approvals of pembrolizumab in the adjuvant setting, ICI-based combination therapies in the 1 L setting, and nivolumab in the second-line (2 L) setting have changed historical treatment paradigms. With this change in the treatment landscape, understanding of real-world treatment patterns and clinical outcomes in the post-ICI setting is critical for informing healthcare decision makers. As newer combination regimens are being studied, it is also important to assess the unmet need based on currently used treatment options and clinical effectiveness outcomes, specifically in patients with mccRCC and disease progression despite receiving treatment for RCC with ICI in the adjuvant, 1 L or 2 L setting. Therefore, the aim of this study was to describe treatment patterns and clinical effectiveness outcomes among patients with mccRCC who received a prior ICI-containing regimen in US community oncology practices.

## Materials and methods

This was a retrospective observational cohort study of adult patients with mccRCC who experienced disease progression on their initial ICI as mono- or combination therapy in adjuvant, 1 L or 2 L settings and received at least one subsequent therapy (index) between 01 January 2018 and 31 January 2023. Patients were included in the study if they had at least 2 visits or one visit with a record of death prior to the end of the observation period (31 July 2023) and excluded if they participated in an interventional clinical trial at any time or received treatment for multiple primary cancer diagnoses following the index date.

Data were sourced from structured fields of The US Oncology Network’s iKnowMed (iKM) electronic health record (EHR) supplemented with chart abstraction to verify additional eligibility criteria (ie, disease progression post-ICI but prior to index) and capture additional unstructured data from a random sample of the population identified from structured data. The US Oncology Network includes over 2700 providers in more than 600 sites of care across the US.^29^ Commercially available datasets that contain death dates sourced from repositories of claims data and obituary records were used to supplement death dates in the EHR.

Descriptive analyses of patient characteristics were presented overall, by lines of therapy (LOT) of the first ICI-containing regimen (adjuvant or 1 L, 2 L) and by index treatment class (IO+IO, IO+TKI, TKI only, other). IMDC risk category was collected as documented in the charts and/or derived from each prognostic indicator with an algorithm applied to assign IMDC risk when possible despite missing data (eg, 1 risk factor and 1 missing = intermediate risk; 3 or more risk factors = poor risk regardless of missingness). Treatment sequencing was presented in Sankey diagrams by LOT of the first ICI. Time-to-event outcomes included time on treatment (ToT), time to next treatment (TTNT), real-world progression-free survival (rwPFS) and overall survival (OS), which were assessed from initiation of the index regimen using unadjusted Kaplan-Meier (K-M) analysis. ToT was defined as the interval between treatment initiation and treatment discontinuation of the index regimen, and TTNT was defined as the interval between the index date and initiation of next treatment after the index date or the date of death due to any cause prior to next treatment. rwPFS was the interval between the index date and the date of disease progression or death. Disease progression was abstracted from the charts as documented in the EHR by the treating clinician based on routine clinical assessment and imaging interpretation, rather than progression determined using standardized RECIST criteria. OS was defined as the interval between the index date and the date of death. Patients without an observed event were censored from the respective analyses on the last contact date prior to the end of the observation period.

## Results

**Figure 1.**
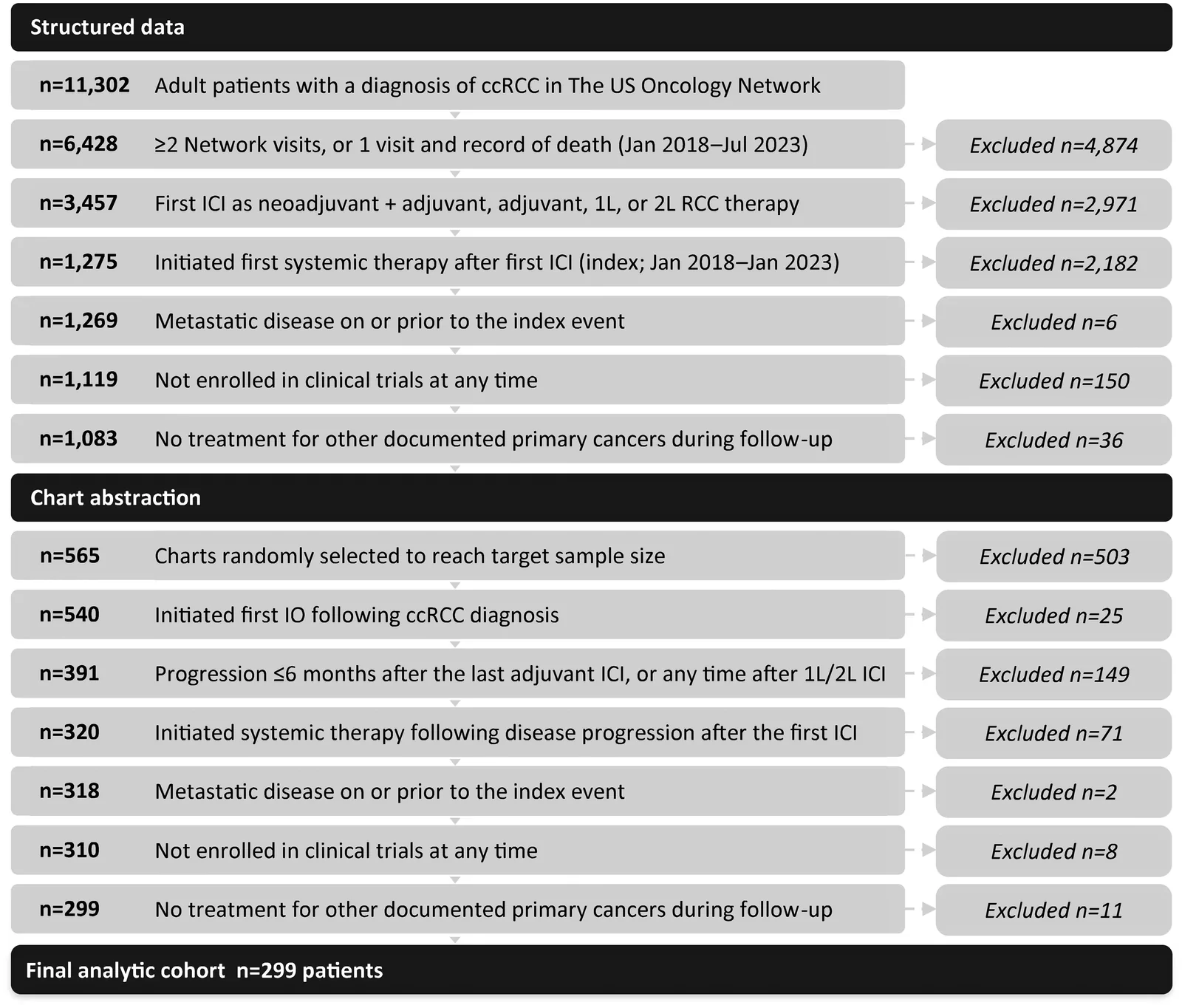
Selection flow diagram. Abbreviations: 1L/2L = first-/second-line systemic therapy for RCC; ccRCC = clear cell renal cell carcinoma; ICI = immune checkpoint inhibitor.

Of 1083 patients that met preliminary eligibility criteria from structured data, 565 patients were screened for eligibility and *N* = 299 eligible patients were included in the study, as shown in Figure 1, with median follow-up of 11.3 months (IQR 5.5-19.6). Demographic and clinical characteristics are described in Table 1. Among patients with available data, median age was 68 years (IQR 60-75), 73% were male, 85% were white, and 87% had an intermediate or poor IMDC risk score. The most common distant metastatic sites were lung (49%), bone (33%), lymph nodes (19%) and liver (13%).

**Table 1.** Baseline demographics and clinical characteristics, overall, by LOT and by index regimen class.

Variable	Overall (*N* = 299)	LOT of 1^st^ ICI**^a^**		Index regimen class (regimen after 1^st^ ICI)**^a^**			
		1L (*N* = 203)	2L (*N* = 91)	TKI Only (*N* = 186)	IO+TKI (*N* = 52)	IO+IO (*N* = 25)	Other (*N* = 28)
**Age at index (years)**							
**Mean (SD)**	66.8 (10.6)	65.9 (10.6)	68.9 (10.5)	67.6 (10.6)	65.0 (11.8)	64.8 (9.5)	66.7 (10.2)
**Median (IQR)**	68 (60–75)	67 (59–74)	70 (62–76)	68 (61–76)	65 (60–73)	65 (59–74)	68 (58–73)
**Sex, n (%)**							
**Male**	217 (72.6)	150 (73.9)	64 (70.3)	133 (71.5)	42 (80.8)	19 (76.0)	18 (64.3)
**Female**	82 (27.4)	53 (26.1)	27 (29.7)	53 (28.5)	10 (19.2)	6 (24.0)	10 (35.7)
**Race, n (%)**							
**White**	222 (74.2)	144 (70.9)	75 (82.4)	145 (78.0)	37 (71.2)	14 (56.0)	21 (75.0)
**Other**	40 (13.4)	30 (14.8)	10 (11.0)	20 (10.8)	8 (15.4)	6 (24.0)	<5
**N/A**	37 (12.4)	29 (14.3)	6 (6.6)	21 (11.3)	7 (13.5)	5 (20.0)	<5
**Practice location, n (%)**							
**South**	101 (33.8)	68 (33.5)	31 (34.1)	63 (33.9)	17 (32.7)	12 (48.0)	5 (17.9)
**Midwest**	94 (31.4)	72 (35.5)	21 (23.1)	55 (29.6)	23 (44.2)	9 (36.0)	6 (21.4)
**West**	96 (32.1)	57 (28.1)	37 (40.7)	62 (33.3)	11 (21.2)	<5	17 (60.7)
**Northeast**	8 (2.7)	6 (3.0)	<5	6 (3.2)	<5	<5	–
**BMI category at index, n (%)**							
**Non-obese**	200 (66.9)	127 (62.6)	69 (75.8)	128 (68.8)	30 (57.7)	19 (76.0)	17 (60.7)
**Obese**	82 (27.4)	62 (30.5)	19 (20.9)	51 (27.4)	18 (34.6)	4 (16.0)	8 (28.6)
**N/A**	17 (5.7)	14 (6.9)	3 (3.3)	7 (3.8)	4 (7.7)	2 (8.0)	3 (10.7)
**Stage at initial RCC diagnosis, n (%)**							
**Stage I-III**	112 (37.5)	63 (31.0)	46 (50.5)	68 (36.6)	16 (30.8)	11 (44.0)	14 (50.0)
**Stage IV**	155 (51.8)	118 (58.1)	35 (38.5)	95 (51.1)	32 (61.5)	10 (40.0)	14 (50.0)
**N/A**	32 (10.7)	22 (10.8)	10 (11.0)	23 (12.4)	4 (7.7)	4 (16.0)	–
**ECOG PS within 30 days of index, n (%)**							
**0-1**	154 (51.5)	103 (50.7)	48 (52.7)	97 (52.2)	26 (50.0)	16 (64.0)	12 (42.9)
**2+**	48 (16.1)	32 (15.8)	15 (16.5)	33 (17.7)	7 (13.5)	1 (4.0)	6 (21.4)
**N/A**	97 (32.4)	68 (33.5)	28 (30.8)	12 (6.5)	1 (1.9)	3 (12.0)	2 (7.1)
**Sarcomatoid features/differentiation at index, n (%)**							
**Yes**	35 (11.7)	25 (12.3)	8 (8.8)	22 (11.8)	7 (13.5)	1 (4.0)	4 (14.3)
**Location of metastatic sites at index, n (%)**							
**Lung**	148 (49.5)	98 (48.3)	47 (51.6)	93 (50.0)	23 (44.2)	17 (68.0)	14 (50.0)
**Bone**	100 (33.4)	75 (36.9)	24 (26.4)	61 (32.8)	21 (40.4)	9 (36.0)	7 (25.0)
**Lymph nodes**	56 (18.7)	39 (19.2)	16 (17.6)	35 (18.8)	6 (11.5)	6 (24.0)	6 (21.4)
**Liver**	39 (13.0)	27 (13.3)	12 (13.2)	22 (11.8)	9 (17.3)	3 (12.0)	3 (10.7)
**Brain**	25 (8.4)	19 (9.4)	6 (6.6)	16 (8.6)	4 (7.7)	1 (4.0)	4 (14.3)
**IMDC risk category at index (no. risk factors), n (%)**							
**Favorable**	33 (11.0)	17 (8.4)	15 (16.5)	20 (10.8)	2 (3.8)	6 (24.0)	4 (14.3)
**Intermediate**	160 (53.5)	118 (58.1)	40 (44.0)	100 (53.8)	33 (63.5)	13 (52.0)	10 (35.7)
**Poor**	69 (23.1)	45 (22.2)	22 (24.2)	40 (21.5)	12 (23.1)	5 (20.0)	10 (35.7)
**N/A**	37 (12.4)	23 (11.3)	14 (15.4)	26 (14.0)	5 (9.6)	1 (4.0)	4 (14.3)
**Nephrectomy prior to index, n (%)**							
**Yes**	201 (63.9)	114 (56.2)	72 (79.1)	118 (63.4)	30 (57.7)	17 (68.0)	19 (67.9)
**No or N/A**	108 (36.1)	89 (43.8)	19 (20.9)	68 (36.6)	22 (42.3)	8 (32.0)	9 (32.1)

Abbreviations: 1L/2L/3L = first-/second-/third-line systemic therapy for RCC; BMI = body mass index, ECOG PS = Eastern Cooperative Oncology Group performance status; IMDC = International Metastatic RCC Database Consortium; IO = immuno-oncology; IQR = interquartile range; N/A = not documented; SD = standard deviation; TKI = tyrosine kinase inhibitor.

a Results for the first ICI in the adjuvant setting (*n* = 5) and ICI monotherapy index regimen class (*n* = 8) subgroups are not displayed due to low sample size.

**Figure 2.**
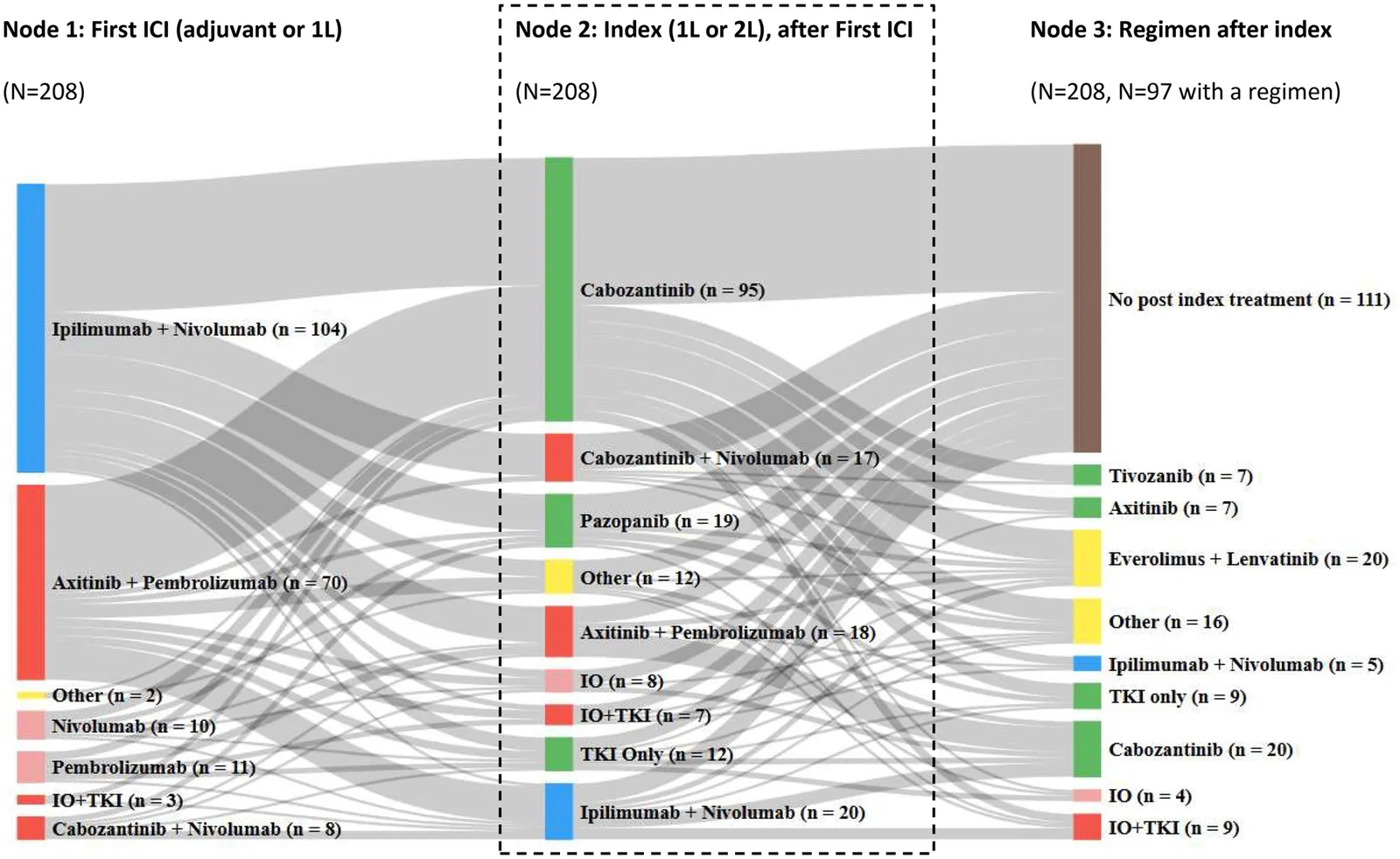
Treatment sequencing among patients who received their first ICI regimen in the adjuvant or 1 L settings. Note: pembrolizumab was initiated as the first ICI treatment in the adjuvant setting in n = 4 patients, and in the 1 L setting after the metastatic diagnosis date in n = 7 patients. Among patients who received first ICI in the adjuvant setting (n = 5), 1L index regimens included cabozantinib, nivolumab + ipilimumab, pembrolizumab + lenvatinib and pazopanib, and regimens after index (n = 2) included axitinib and nivolumab + ipilimumab. Of n = 111 patients with no post-index treatment, 59% (n = 65) died and 32% (n = 35) had ongoing treatment.

**Figure 3.**
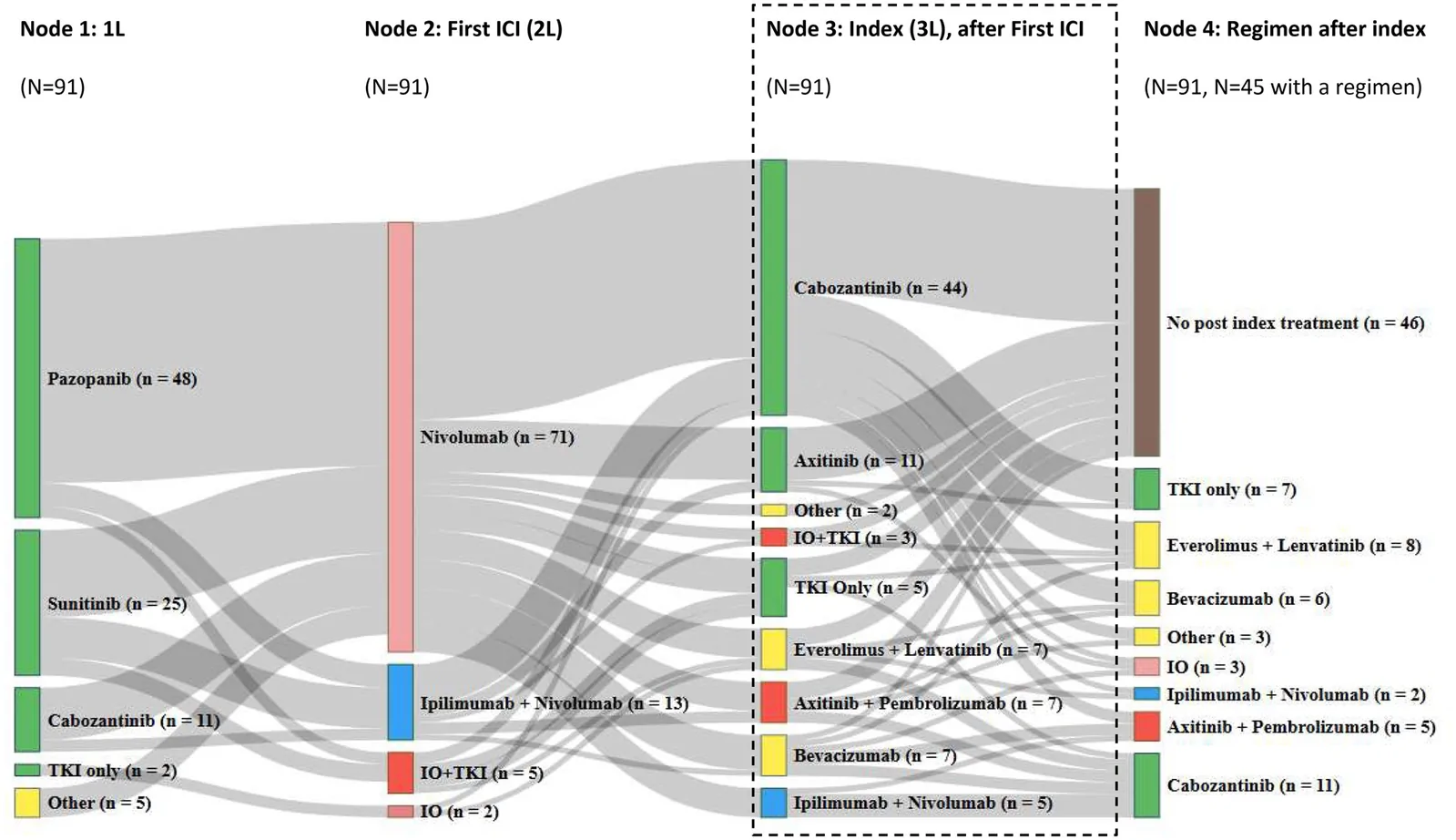
Treatment sequencing among patients who received their first ICI regimen in 2 L setting. Note: Of n = 46 patients with no post-index treatment, 70% (n = 32) died and 22% (n = 10) had ongoing treatment.

Of 299 patients included in the cohort, *n* = 5 (2%), *n* = 203 (68%), and *n* = 91 (30%) received first ICI-containing regimen in the adjuvant, 1 L and 2 L settings, respectively. The distribution and sequencing of treatment regimens among patients who received their first ICI regimen in the adjuvant/1L or 2 L settings are presented in Figures 2 and 3, respectively. Among patients who initiated ICI-containing regimen in the 1 L setting (*n* = 203), the most commonly used 1 L regimen was nivolumab+ipilimumab (*n* = 103, 51%), followed by pembrolizumab+axitinib (*n* = 70, 34%). Among patients who initiated the first ICI-containing regimen in the 2 L setting (*n* = 91), the most commonly used 2 L therapy was nivolumab (*n* = 71, 78%), whereas 14% (*n* = 13) received 2 L nivolumab+ipilimumab. In these patients, 1 L TKI monotherapy was the most common regimen class prior to ICI (pazopanib *n* = 48, 53%; sunitinib *n* = 25, 27%; cabozantinib *n* = 11, 12%). TKI monotherapy was also the most common index treatment across patients who initiated a first ICI-containing regimen in 1 L or 2 L settings (cabozantinib: 46%; pazopanib: 7%; axitinib: 7%; other TKI: 2%), followed by IO+TKI (pembrolizumab+axitinib: 9%; nivolumab+cabozantinib: 6%; other IO+TKI: 3%), IO+IO (nivolumab+ipilimumab: 8%), other regimens (everolimus+lenvatinib: 4%; bevacizumab: 2%; other: 3%) and IO monotherapy (3%).

Overall, the median (IQR) time from first ICI treatment initiation to start of the index regimen was 6.7 (3.4-13.0) months. From initiation of the index regimen, the median (95% CI) ToT was 5.5 (4.8-6.2) months. Around half of the patients (*n* = 142, 47%) proceeded to the next regimen, 97 (32.4%) did not proceed to a next regimen and died, and an additional 15 (5%) patients discontinued treatment, did not proceed to a next regimen, and had no record of death during the observation period. Median (95% CI) TTNT was 7.1 (6.2-8.9) months and the most common treatments after index among patients who proceeded to a next regimen (*n* = 142) were cabozantinib (*n* = 31, 22%), everolimus+lenvatinib (*n* = 28, 20%) and bevacizumab (*n* = 11, 8%).

**Figure 4.**
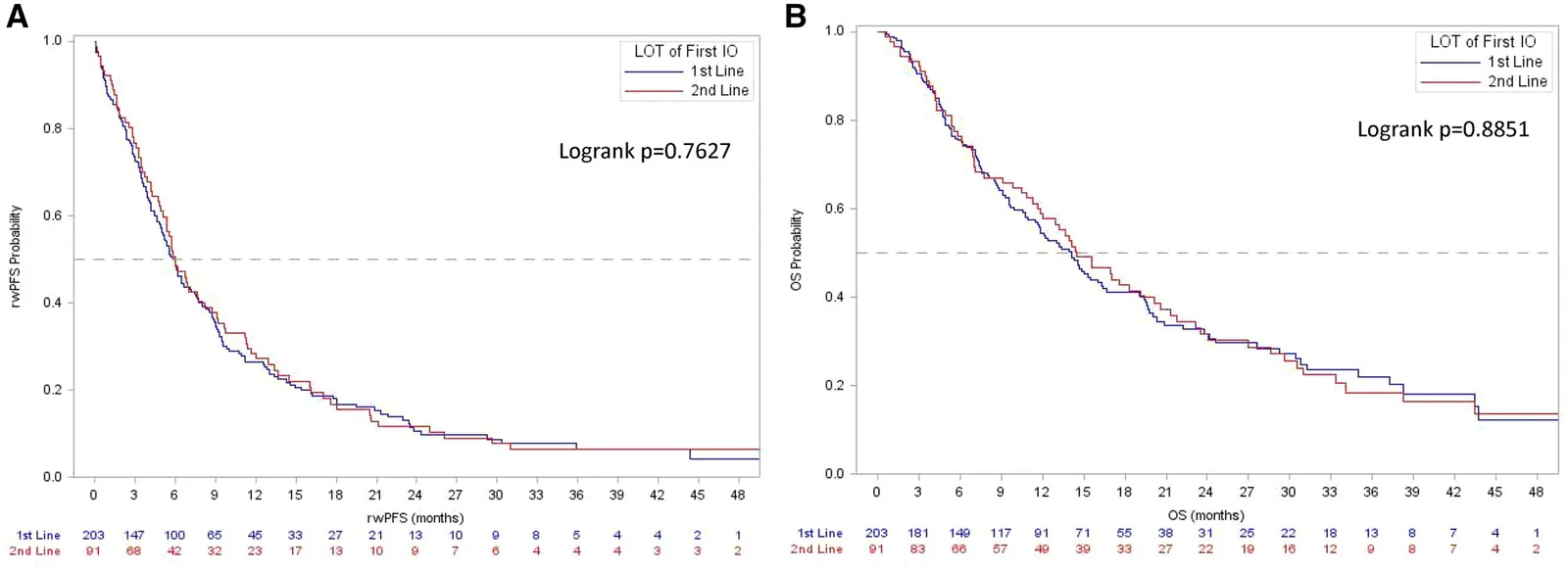
Survival curves of (A) rwPFS and (B) OS from index by LOT of the first IO. Note: results for the adjuvant → 1 L (n = 5) subgroup are not displayed due to low sample size.

**Figure 5.**
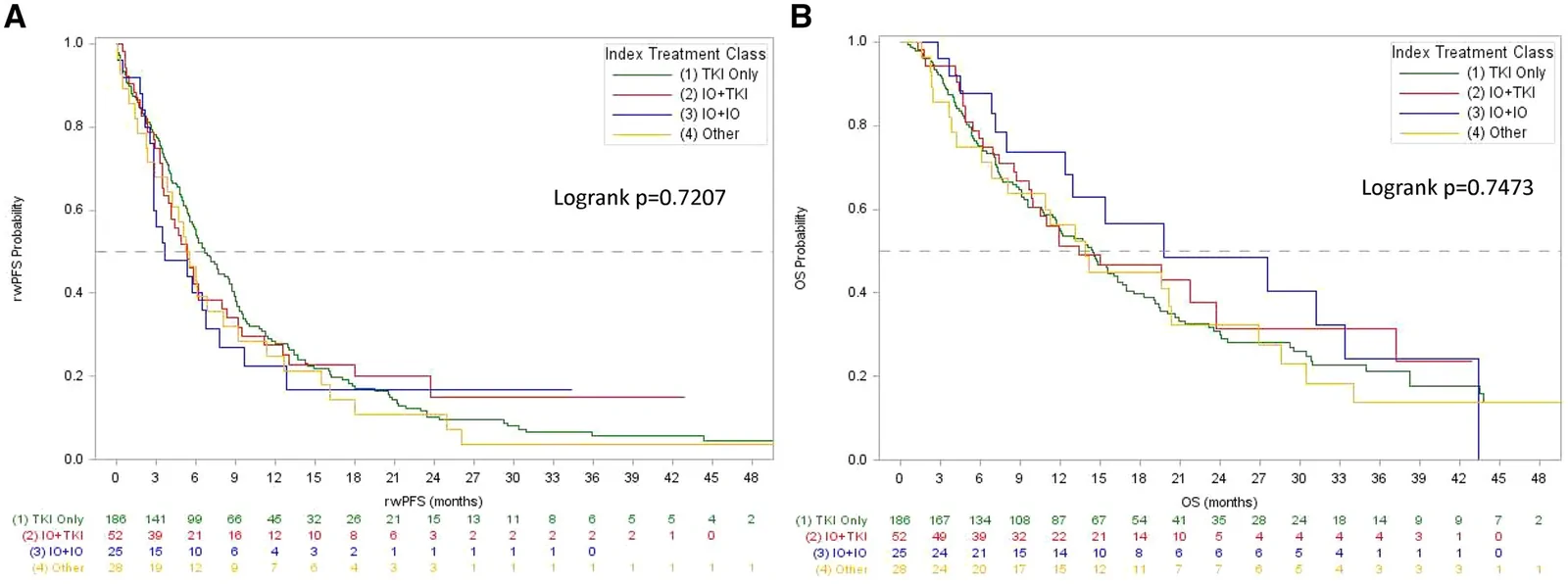
Survival curves of (A) rwPFS and (B) OS from index by treatment class. Note: results for the ICI monotherapy (n = 8) subgroup are not displayed due to low sample size.

Results from the unadjusted analyses of clinical outcomes are presented in Figures 4 and 5 and Table S1. Overall, the median (95% CI) rwPFS from initiation of the post-ICI index regimen were 6.0 (5.4-6.8) months and the 12-month (95% CI) rwPFS probability was 26.8% (21.7%-32.1%). The median (95% CI) OS was 14.3 (12.1-16.4) months and the 12-month (95% CI) OS probability was 56.1% (50.0%-61.7%). There were no statistically significant differences observed in the unadjusted analyses of rwPFS or OS by LOT of the first ICI (rwPFS logrank *P* = 0.763; OS *P* = 0.885) or index treatment class (rwPFS *P* = 0.721; OS *P* = 0.747).

## Discussion

This retrospective, observational study evaluated demographic and clinical characteristics, treatment patterns and clinical outcomes of real-world patients (*N* = 299) diagnosed with mccRCC who initiated a subsequent treatment (index) following disease progression after an initial ICI-containing regimen. VEGFR-TKI monotherapy was the most common index regimen class for both patients who initiated ICI in 1 L (*n* = 123, 61%) or 2 L (*n* = 60, 66%) settings. ICI-based combinations (IO+IO or IO+TKI) were given as the post-index regimen in 30% (*n* = 60) and 16% (*n* = 15) of patients who initiated ICI in 1 L and 2 L, respectively. The most common index regimen was cabozantinib monotherapy (*n* = 93, 46% for patients who initiated ICI in 1 L and *n* = 44, 48% for patients who initiated ICI in 2 L). While nearly half of patients proceeded to a post-index regimen during the observation period (*n* = 97, 47% for patients who initiated ICI in adjuvant or 1 L, and *n* = 45, 49% for patients who initiated ICI in 2 L), almost a third of patients did not proceed to a subsequent treatment and died during the study period underscoring the need for effective treatment options in the post-ICI setting. Overall, median (95% CI) rwPFS and OS from initiation of the index regimen were 6.0 (5.4-6.8) and 14.3 (12.1-16.4) months, respectively. While approximately two-thirds (68%) of the cohort received the first ICI in the 1 L setting (mostly nivolumab+ipilimumab or pembrolizumab+axitinib), nearly one-third initiated the first ICI in 2 L (mostly nivolumab monotherapy) which was within expectations considering the evolution in clinical practice throughout the study identification period (January 2018 to January 2023). Significant differences in rwPFS and OS from index in patients initiating ICI in 1 L vs. 2 L were not observed, though these groups represent distinct populations as evidenced by differences in patient characteristics such as stage IV at initial diagnosis (58% vs. 38%), sarcomatoid features (12% vs. 9%), and metastatic sites (bone: 37% vs. 26%; brain: 9% vs. 6%).

Treatment patterns and clinical outcomes observed in this study are consistent and/or within expectations based on findings from other real-world studies of similar patient populations. Shah et al., 2019^30^ conducted a retrospective study of 70 patients treated with 2 L VEGFR-TKI after progressive disease with 1 L IO.^30^ The most common 1 L regimens included nivolumab+ipilimumab (47%), IO+bevacizumab (36%) and ICI monotherapy (17%). The median (95% CI) PFS from initiation of the post-ICI regimen was 13.2 (10.1-NR) months and 41% (*n* = 28) had a complete or partial response to treatment. Ged et al., 2020 conducted a retrospective analysis of 59 patients treated post-discontinuation of IO+VEGF regimens.^31^ The most common subsequent therapies included cabozantinib (*n* = 22; 37%), axitinib (*n* = 18; 31%) and pazopanib (*n* = 4; 7%), and 71% of patients received ICI in the 1 L setting. While the median PFS and OS were 12.0 and 24.5 months, respectively, the overall response rate was 25%. A subset of patients (*n* = 66) from Geynisman et al., 2021 provided information on real-world treatment patterns following 1 L nivolumab+ipilimumab.^32^ The most common 2 L regimens were cabozantinib (*n* = 33, 50%) and pazopanib (*n* = 8, 12%), and 22 (33%) patients proceeded to 3 L including axitinib and everolimus+lenvatinib (*n* = 4, 18%, each). El Zarif et al., 2024 evaluated treatment patterns and outcomes in patients previously treated with ICI in the adjuvant setting (*n* = 37, pembrolizumab; *n* = 28, atezolizumab; *n* = 15, nivolumab+ipilimumab) observing 18-month PFS and OS rates of 45% and 85%, respectively.^33^ Lastly, 2 subsets of patients with treatment-refractory advanced RCC from Navani et al., 2023 were treated with 2 L cabozantinib after 1 L IO+VEGF (*n* = 46) and 1 L nivolumab+ipilimumab (*n* = 78), respectively.^34^ Median (95% CI) OS from initiation of 2 L cabozantinib in these cohorts were 15.7 (9.3-NR) and 21.4 (12.1-NR) months, respectively. While these historical, real-world post-ICI cohorts may not all be directly comparable to more contemporary populations which are increasingly exposed to ICI-TKI combinations in 1 L, consistency in the directionality of results with other RWE studies of patients treated for mccRCC in the post-ICI setting also provides further confidence to the results of this study. Furthermore, our study adds substantial value to the existing literature given that it includes a relatively larger sample of patients, does not restrict to specific IO/post-ICI treatments, and is more inclusive around the number of prior treatment regimens.

While there are some notable differences between this real-world study and clinical trials that have looked at similar patient populations, results from this study are also broadly within expectations. The Phase 3 LITESPARK-011 study, which evaluated belzutifan+lenvatinib (*n* = 371) vs. cabozantinib (*n* = 376) in patients with advanced ccRCC that progressed on or after 1 L or 2 L ICI or within 6 months of the last dose of adjuvant ICI therapy, reported median PFS of 10.7-14.8 months and median OS of 27.6-34.9 months across the respective cohorts.^35^ The Phase 3 LITESPARK‑005 study evaluated belzutifan (*N* = 374) vs. everolimus (*N* = 372) in patients with advanced ccRCC previously treated with both an ICI and a VEGFR‑TKI.^27^ Median PFS was 5.6 months in both arms, while median OS ranged from 18.1 to 21.4 months across treatment groups.^27^ The Phase 3 CONTACT-03 study evaluated atezolizumab+cabozantinib (*N* = 263) vs. cabozantinib monotherapy (*N* = 259) in patients with advanced RCC whose disease had progressed with IO. The median OS was 26 months-NR (not reached) and median PFS was 10.6-10.8 months.^23^ The Phase 3 TiNivo-2 study assessed tivozanib+nivolumab (*N* = 171) vs. tivozanib monotherapy (*N* = 162) in patients with advanced RCC and progression during or after 1-2 previous LOT including an IO. For this study, the median PFS was 5.7-7.4 months and median OS was 17.7-22.1.^24^ The Phase 2 FRACTION-RCC study, which evaluated nivolumab+ipilimumab (*N* = 46) along with other ICI combinations (not reported) in patients with advanced RCC that progressed during or after a prior ICI-containing regimen, showed a median PFS and OS and PFS of 3.7 and 23.8 months, respectively.^36^ The Phase 2 LenCabo study, which compared lenvatinib+everolimus (*n* = 40) vs. cabozantinib (*n* = 46) in patients with advanced ccRCC previously treated with PD‑1/PD‑L1 inhibitors, reported a median PFS of 15.7 vs. 10.2 months, respectively.^26^ In the Phase 1/2 KEYMAKER-U03 Substudy 03B, patients with previously treated advanced ccRCC received pembrolizumab+belzutifan (*N* = 62), lenvatinib+belzutifan (*N* = 64), or pembrolizumab+lenvatinib (*N* = 73). Median PFS ranged from 5.4 to 12.5 months, and median OS ranged from 32.3 to NR.^37^ Study 111/KEYNOTE-146, a Phase 2 study of pembrolizumab+lenvatinib that included an RCC cohort and IO-pretreated subgroup (*n* = 104), reported a median PFS of 12.2 months (median OS: NR).^20^ While rwPFS observed in this real-world study is within the range of PFS presented in these clinical trials, it is noted that median OS is longer in each of the RCTs relative to this real-world study but within expectations given the stricter inclusion criteria for RCTs, differences in patient characteristics (ie, ECOG performance status) and study design (ie, follow-up, monitoring).

Due to the study’s observational design, the descriptive outcomes results were susceptible to confounding by baseline characteristics across index regimen class as adjusted analyses were not performed. Additionally, patients with a first ICI-containing regimen in 2 L survived and maintained sufficient clinical fitness to receive multiple lines of therapy, introducing potential survivor and selection biases that may have attenuated differences in outcomes between groups independent of treatment effect. The iKM EHR database is also not collected for research purposes but for clinical practice reasons which may introduce misclassification bias, although the missingness for variables like ECOG status are concordant with other RWD data sources and an algorithm was implemented to derive IMDC risk when not documented to limit missingness. More detailed prognostic measures, such as the Meet-URO score, were not available for evaluation, and may offer more accurate prognostic stratification than IMDC risk classification alone. Several criteria are considered for this study to identify a similar population as observed in post-ICI clinical trials, however, certain clinical trial criteria were not applied to better represent the real-world patient population.^25^ As such, results from this observational study may not be directly comparable to the clinical trial. This study also included few patients treated with ICI in the adjuvant setting, reflecting limited RWD for this increasingly common treatment paradigm and highlighting the need for future studies in this space. In addition, belzutifan was not represented among post‑ICI regimens in the current analysis, underscoring the importance of future RWE studies incorporating novel mechanisms of action as they become more widely used. Not all community oncology practices are included in The US Oncology Network and the results of this study will be most generalizable to other community oncology practices that adhere to evidence-based treatment guidelines.

## Conclusion

This real-world study provides insight on patient characteristics, treatment patterns and clinical outcomes of patients diagnosed with mccRCC who received systemic therapy following disease progression on an ICI-containing regimen. Most patients received immune checkpoint inhibitor (ICI)-based therapy in the first‑line setting, predominantly as nivolumab plus ipilimumab or pembrolizumab plus axitinib, and the majority of these patients proceeded to subsequent therapy, most commonly VEGFR‑TKI monotherapy, particularly cabozantinib. However, unadjusted median rwPFS and OS with post‑ICI VEGFR‑TKI therapy were modest with no statistically significant differences observed between cabozantinib or other VEGFR-TKI, and nearly a third of patients did not proceed to a subsequent regimen post-index and died during the study period. A substantial proportion of patients also initiated the first ICI-containing regimen in the 2 L setting following 1 L VEGFR‑TKI treatment and subsequently received VEGFR-TKI again as the index treatment, yet substantial differences in clinical outcomes were not observed across treatment classes and lines of ICI exposure. Although significant progress has been made in different mccRCC settings, there remains an unmet medical need for innovative and novel therapies in the post-ICI space to improve clinical outcomes, and findings from this real-world study may inform the design of future studies to address this need.

## Supplementary Material

oyag326_Supplementary_Data

## Data Availability

The data underlying this article cannot be shared publicly due to patient privacy considerations and restrictions associated with the use of de-identified electronic health record data from a proprietary database. The data may be shared on reasonable request to the corresponding author, subject to applicable data use agreements and institutional approvals.
