# Supplementary material for: Real-world treatment patterns and outcomes in metastatic clear cell renal cell carcinoma following immune checkpoint inhibitor progression (2018-2023)
Source: Oncologist. 2026 Sep 15;31(10):oyag326. doi: 10.1093/oncolo/oyag326 (PMC13596056; doi:10.1093/oncolo/oyag326)
Supplement: Supplementary file 1 [file oyag326_supplementary_data.docx]

| **Variable** | **Overall**  **(N=299)** | **LOT of 1^st^ ICI^a^** | | **Index regimen class (regimen after 1^st^ ICI)^a^** | | | |
| --- | --- | --- | --- | --- | --- | --- | --- |
|  |  | **1L**  **(N=203)** | **2L**  **(N=91)** | **TKI Only**  **(N=186)** | **IO+TKI**  **(N=52)** | **IO+IO**  **(N=25)** | **Other**  **(N=28)** |
| **rwPFS** | | | | | | | |
| Events, n (%) | 256 (85.6) | 173 (85.2) | 80 (87.9) | 161 (86.6) | 41 (78.8) | 20 (80.0) | 27 (96.4) |
| Median (95% CI), months | 6.0 (5.4,6.8) | 6.0 (5.0,7.1) | 6.0 (5.1,8.2) | 6.7 (5.6,8.5) | 5.4 (3.6,8.0) | 3.6 (2.8,6.7) | 5.4 (2.9,8.0) |
| IQR, months | 2.9,13.0 | 2.8,12.9 | 3.3,13.4 | 3.4,13.7 | 3.1,13.0 | 2.8,9.7 | 2.3,12.0 |
| Probability, % (95% CI) | | | | | | | |
| 6 months | 50.1 (44.3,55.7) | 50.1 (43.0,56.8) | 49.5 (38.7,59.4) | 55.5 (47.9,62.3) | 42.3 (28.8,55.2) | 40.0 (21.3,58.1) | 42.9 (24.6,60.0) |
| 12 months | 26.8 (21.7,32.1) | 26.5 (20.5,33.0) | 27.1 (18.2,36.7) | 27.7 (21.3,34.6) | 27.5 (16.0,40.3) | 22.5 (8.5,40.5) | 25.0 (11.1,41.8) |
| 18 months | 17.7 (13.4,22.6) | 18.0 (12.8,24.0) | 16.8 (9.7,25.6) | 17.7 (12.3,24.0) | 22.9 (12.3,35.5) | 16.9 (4.9,35.0) | 14.3 (4.5,29.5) |
| 24 months | 11.1 (7.5,15.5) | 10.5 (6.3,16.0) | 11.6 (5.8,19.7) | 10.2 (6.1,15.6) | 15.0 (5.5,29.1) | 16.9 (4.9,35.0) | 10.7 (2.7,25.1) |
| **OS** | | | | | | | |
| Events, n (%) | 198 (66.2) | 131 (64.5) | 67 (73.6) | 125 (67.2) | 30 (57.7) | 14 (56.0) | 22 (78.6) |
| Median (95% CI), months | 14.3 (12.1,16.4) | 14.1 (11.5,16.3) | 14.5 (11.6,20.1) | 14.4 (11.6,16.7) | 13.4 (9.6,23.8) | 19.7 (12.4,33.3) | 13.9 (6.9,26.9) |
| IQR, months | 6.2,30.8 | 6.1,30.8 | 6.2,30.5 | 6.1,30.5 | 6.2,37.2 | 8.0,33.3 | 5.1,28.6 |
| Probability, % (95% CI) | | | | | | | |
| 6 months | 76.2 (70.9,80.6) | 75.4 (68.8,80.8) | 76.5 (66.2,84.0) | 75.2 (68.2,80.8) | 76.9 (63.0,86.2) | 87.7 (66.4,95.8) | 75.0 (54.6,87.2) |
| 12 months | 56.1 (50.0,61.7) | 54.6 (47.1,61.4) | 57.7 (46.6,67.3) | 54.9 (47.1,62.0) | 51.4 (36.5,64.4) | 73.5 (50.1,87.2) | 56.3 (35.9,72.4) |
| 18 months | 42.1 (36.0,48.0) | 41.2 (33.8,48.4) | 42.7 (32.0,53.0) | 39.7 (32.1,47.2) | 46.7 (32.1,60.0) | 56.7 (32.5,75.1) | 45.0 (26.0,62.3) |
| 24 months | 31.9 (25.9,38.0) | 31.7 (24.4,39.3) | 31.7 (21.8,42.0) | 30.8 (23.5,38.4) | 31.4 (15.3,49.0) | 48.6 (24.0,69.4) | 32.2 (15.5,50.3) |
